# Evaluating the impact of loneliness and social isolation on health literacy and health-related factors in young adults

**DOI:** 10.3389/fpsyg.2023.996611

**Published:** 2023-01-27

**Authors:** Shradha Vasan, Nina Eikelis, Michelle H. Lim, Elisabeth Lambert

**Affiliations:** Iverson Health Innovation Research Institute, Swinburne University of Technology, Hawthorn, VIC, Australia

**Keywords:** loneliness, health-related factors, health literacy, social isolation, young adults

## Abstract

**Objectives:**

In current study, we aim to extend previous research by investigating the unique impact of loneliness on health literacy and health-related factors of young adults, after controlling for social isolation, depressive symptomology, and social anxiety, as well as evaluate how social isolation and loneliness differ in their impact on health literacy, and health-related factors among young adults, after accounting for abovementioned concomitant variables.

**Methods:**

Using a cross-sectional study design, 521 young adults completed an online survey in 2020, where they self-reported their loneliness, social isolation, health-related factors, and health literacy data.

**Results:**

Increased loneliness was associated with decrease in several health literacy domains (e.g., poorer social support for health, lower appraisal of health information, among others) and increase in some health-related factors (e.g., higher perceived stress, higher negative affect), among young adults, even after controlling for social anxiety, depressive symptomology, and social isolation. Contrastingly, increase in social isolation was associated with changes in some health-related factors - more somatic health complaints, higher alcohol use, poorer cognitive and physical functioning, and lower scores for only one health literacy domain (i.e., social support for health) among young adults, after adjusting for the influence of social anxiety, depressive symptomology, and loneliness.

**Conclusion:**

Even after accounting for the influence of several co-occurring social and mental health symptoms, higher loneliness was associated poorer health literacy and health-related factors in young adults. We also found loneliness and social isolation may differ in the mechanisms through which they impact health literacy and health-related factors in young adults.

## Introduction

Young adulthood is a critical and transitional period marked with psychosocial challenges (e.g., striving for independence, holding increased responsibilities, and moving away from familial relationships and gravitating toward peer-based relationships; Heinrich and Gullone, 2006). As such, young adults (<35 years old) are susceptible to adverse mental health experiences, particularly feelings of loneliness (Lim et al., 2019; Cigna, 2020; Negosanu and Reid, 2021; Varga et al., 2021). Loneliness is a subjective and adverse experience which arises due to differences between an individual’s actual and desired quality of social relationships (Peplau, 1982). It is more closely associated with the quality of social interactions rather than the quantity or number of social contacts of an individual (de Jong and Havens, 2004). Loneliness is a relatively common experience. Indeed, a 2018 survey found 51 percent of Australian adults felt lonely at least one day a week (Abbott et al., 2018). Most researchers tend to focus on understanding the impact of loneliness on older adults [i.e., >65 years old; (Pinquart and Sorensen, 2001; Routasalo and Pitkala, 2003; Cohen-Mansfield et al., 2016; Kemperman et al., 2019)]. Nonetheless, recent studies show loneliness follows a bi-modal, U-shaped distribution (Yang and Victor, 2011; Pyle and Evans, 2018), where feelings of loneliness peak during adolescence and young adulthood (<35 years), recede in middle adulthood, and increase again in older adulthood [>70 years; (Hawkley et al., 2010; Lasgaard et al., 2016)]. Findings from these studies clearly demonstrate that young adults are just as susceptible to loneliness as older adults (Lim et al., 2019; Cigna, 2020; Negosanu and Reid, 2021; Varga et al., 2021).

Loneliness has been globally recognized as a growing public health concern with a detrimental impact on health (Hunter, 2012; Lim et al., 2020). Findings from Holt-Lunstad et al. (2015) meta-analytic review argues experience of loneliness increases the likelihood of mortality by 26%. Robust evidence from cross sectional and longitudinal research demonstrates the negative impact loneliness can have on physical health [i.e., higher incidences of cardiovascular issues (Grant et al., 2009; Thurston and Kubzansky, 2009)], cognitive health (Shankar et al., 2013), and mental health [i.e., associated with anxiety disorders (Beutel et al., 2017)], psychosis (Sündermann et al., 2014; Lim et al., 2018), schizophrenia (Trémeau et al., 2016), suicidality (Stickley and Koyanagi, 2016), paranoia (Sündermann et al., 2014), social anxiety (Lim et al., 2016), and depression (Cacioppo et al., 2010). For comprehensive review on the impact of loneliness on health, see (Hawkley and Cacioppo, 2010; Lim et al., 2020).

There are several pathways through which loneliness may affect health of young adults, including health literacy and health-related factors. Health literacy refers to the application of social and cognitive skills needed to access, assess, and engage with health-related information to support and sustain good health, and make informed health-related decisions (Nutbeam, 1998). An extensive literature documents the association between lower health literacy and poorer health outcomes (Marvanova et al., 2011; Mitchell et al., 2012; Geboers et al., 2016; Miller, 2016). Further, limited research indicates an inverse relationship between loneliness and health (Bennett et al., 2012; Geboers et al., 2016; Vasan et al., 2022). In the current study, health-related factors are comprised of several physical health factors (e.g., body mass index (BMI), weight, diet, sleep, somatic health complaints, alcohol use, physical activity, perceived general health, cognitive and physical functioning), and psychosocial factors (e.g., quality of life, perceived stress, positive affect, and negative affect). There is some evidence to suggest a negative relationship between loneliness and some of these health-related factors. Higher loneliness is associated with higher obesity (Fernández-Alonso et al., 2012; Hajek and König, 2019), elevated BMI (Fernández-Alonso et al., 2012), lower quality of life (Ong et al., 2016; Khalaila and Vitman-Schorr, 2018) among older adults. Loneliness is associated with increased sleep difficulties [e.g., poor sleep quality, sleep fragmentation/disturbances, poor day time functioning; (Hawkley et al., 2010; Kurina et al., 2011; Eccles et al., 2020; Griffin et al., 2020; Shankar, 2020)], and overall poorer self-rated health (Richard et al., 2017; Counts and John-Henderson, 2020; Eccles et al., 2020; Marziali et al., 2020; Christiansen et al., 2021). Researchers have found mixed results for the effect of loneliness on physical activity, with Hawkley and colleagues (Hawkley et al., 2009; Luo and Waite, 2014) demonstrating higher loneliness to be associated with lower levels of physical activity. Conversely, studies have found no impact of loneliness on physical activity levels (McKee et al., 2015). There is some evidence to suggest a positive relationship between loneliness and somatic health issues (e.g., asthma, allergy, diabetes, migraine, osteoarthritis) in adolescence and young adults (Stickley et al., 2016; Christiansen et al., 2021). Increased experience of loneliness is positively correlated with perceived stress and positive affect among college students (Counts and John-Henderson, 2020). Likewise, negative relationships have been reported between loneliness and negative affect across different age groups (Kurina et al., 2011; Ijzerman et al., 2012; Counts and John-Henderson, 2020). Taken together, these findings demonstrate the detrimental impact feelings of loneliness can have on various health-related factors and to some extent, health literacy. Nonetheless, aforementioned studies are not without limitations. The main issues identified in existing literature are, as mentioned previously, limited focus on the impact of loneliness on health literacy and health-related factors in young adults, especially in Australia, and the oversight to account for the influence of other co-occurring social and mental health symptoms such as, social isolation, depressive symptomology, and social anxiety.

The relationship between loneliness and several social and mental health issues (i.e., social isolation, depressive symptomology, and social anxiety) is nuanced and multifaceted (Christiansen et al., 2021). It is well established that depression has a detrimental impact on health in general (Belvederi Murri et al., 2018). However, recent research has identified loneliness as a potential antecedent for depression (Cacioppo et al., 2010). Further, loneliness has a more reciprocal or bidirectional relationship with social anxiety. Lim et al., (2016) found baseline loneliness predicted social anxiety, paranoia, and depression when measured over a six-month period in a community sample. However, only social anxiety predicted loneliness above and beyond paranoia and depression (Lim et al., 2016) [for a review, see (Maes et al., 2019)]. Further, in the present study, we have given special attention to the influence of social isolation and its relationship with loneliness. Often, the construct of social isolation is interchangeably and synonymously used with the term loneliness (de Jong and Havens, 2004) [for examples, see (Caspi et al., 2006; Banerjee and Rai, 2020)]. It is important to understand that while related, social isolation and loneliness are distinct concepts (Cornwell and Waite, 2009). Loneliness is a subjective emotional experience *where one feels their current relationships do not meet their desired social need*, conversely, social isolation is an objective, quantifiable variable which measures the number of an individual’s social interactions and social contacts (Asher and Paquette, 2003; de Jong and Havens, 2004). Indeed, in a recent study Beller and Wagner, (2018) found loneliness and social isolation differ significantly in how they impact mortality among older adults. Therefore, to capture the *unique* influence of loneliness on health literacy and health-related factors, especially in a less researched population such as young adults, it is crucial to consider the role of these co-occurring social and mental health issues.

The aims of the present cross-sectional study are: (1) to extend previous research by investigating the unique influence of loneliness on health literacy and health-related factors among young adults (<35 years old), after controlling for social isolation, depressive symptomology, and social anxiety. It is hypothesized that higher loneliness in young adults would predict lower health literacy and poorer responses on health-related factors, after controlling for social isolation, depressive symptomology, and social anxiety. (2) to evaluate how social isolation (a term sometimes used interchangeably with loneliness), and loneliness may differ in their impact on health-related factors and health literacy of young adults. It is hypothesized that loneliness and social isolation would differ in their associations with health- related factors and health literacy domains in young adults.

## Methods

Ethical approval was granted by local Australian University’s ethics committee. The data used in the current study was a subset of a larger study. Participants completed a self-report survey hosted on Qualtrics (survey platform). Survey was distributed through word of mouth, advertisements on social networks, online forums, and *via* research training program for undergraduate first year students. Participation was voluntary, explanatory statement was included in the survey, and informed consent was obtained prior to participants completing the survey. All data was deidentified to maintain participants privacy and confidentiality. Data were collected between June 2020 and November 2020. A total of 521 Australian young adults were included in the final dataset. The online survey included demographic questions, comprehensive measures for loneliness, social isolation, social anxiety, and depressive symptomology. Further, a health literacy scale, and measures for different health-related factors were also included. Details for all scales and materials used in the current study are as follows.

### Materials

Demographic information including age, gender, use of English as primary language, marital status, ethnicity, sexual orientation, education level, living status, chronic conditions, and smoking status were obtained. Participants self-reported their weight and height measurements as well as number of diagnosed chronic conditions. BMI scores were calculated from the self-reported weight and height measurements. Scores for physical activity and alcohol use were calculated using participant’s responses for frequency and intensity of use. Additional information on how these abovementioned health-related outcomes were measured is provided in Supplementary material. The internal reliability values (Cronbach’s *α*) for the data and scales used in the present study are included in Table 1.

**Table 1:** Descriptive statistics and internal reliability (Cronbach’s *α*) scores.

Scale	Mean score	SD	Possible scores range (Lowest-Highest)	Actual score range (Lowest-Highest)	Cronbach’s *α*
Health-related factors					
Weight	70.43	14.62	–	35–125	–
Body mass index	24.76	4.72	–	14–43	–
Perceived general health	2.96	0.93	1–5	1–5	–
Somatic health	44.30	12.02	7–98	14–82	0.85
Sleep	11.34	5.69	0–28	0–28	0.88
Diet	66.69	15.83	0–130	12–115	–
Physical activity	7.11	3.2	0–21	0–18	–
Alcohol use	5.38	6.30	0–22	0–22	–
Cognitive and physical functioning	23.56	6.0	7–42	7–41	0.91
Perceived stress	7.89	2.64	0–16	0–16	0.72
Quality of life	27.54	5.08	8–40	11–40	0.85
Positive affect	13.45	3.76	5–25	5–25	0.85
Negative affect	12.22	4.30	5–25	5–25	0.88
Loneliness	47.08	9.60	20–80	20–77	0.93
Social anxiety	26.68	13.00	0–68	0–64	0.95
Social isolation	14.81	4.96	0–30	0–30	0.84
Depression	24.62	11.14	0–60	0–58	0.93
Health literacy scales					
Feeling understood and supported by healthcare providers	2.99	0.62	1.0–4.0	1.0–4.0	0.94
Having sufficient information to manage my health	3.01	0.47	1.0–4.0	1.75–4.0	0.94
Actively managing my health	2.91	0.56	1.0–4.0	1.2–4.0	0.94
Social support for health	2.99	0.58	1.0–4.0	1.0–4.0	0.94
Appraisal of health information	2.93	0.51	1.0–4.0	1.4–4.0	0.94
Ability to actively engage with healthcare providers	3.73	0.67	1.0–5.0	2.0–5.0	0.96
Navigating the healthcare system	3.70	0.64	1.0–5.0	2.0–5.0	0.96
Ability to find good health information	3.88	0.56	1.0–5.0	2.0–5.0	0.96
Understand health information well enough to know what to do	4.0	0.58	1.0–5.0	2.0–5.0	0.96

*N* = 521. Descriptive statistics and reliability testing (Cronbach’s *α*) scores. Scales without Cronbach’s *α* were either single item measures (i.e., Weight, BMI) or had a different scoring/anchor points for items in the questionnaire (i.e., Diet, physical activity, alcohol use). As the Health literacy questionnaire has two scale points (1 to 4 and 1 to 5), reliability statistics for both the scale points were calculated separately. Possible scores are the theoretical range provided by the author of the original scale; the actual scores are ranges observed in the current study.

#### University of California Los Angeles (UCLA) loneliness scale

The UCLA is a 20-question questionnaire using a 1 (*Never*) to 4 (*Always*) Likert-type scale. UCLA assesses levels of loneliness over a one-month period (Russell, 1996). It includes both positively worded items (e.g., “*How often do you feel that you are “in tune” with the people around you?*”) and negatively worded (e.g., “*How often do you feel that people are around you but not with you?*”) questions (Russell, 1996). Total scores are calculated by reverse coding the positively worded questions and then summing the scores for all the questions, scores range from 20 to 80 with higher scores indicating higher levels of loneliness. The UCLA has been researched extensively across different populations and has demonstrated excellent reliability and convergent validity with related constructs (Russell, 1996).

#### Lubben social network scale

The Lubben social network scale (LSNS) is a 6 item self-reported scale that assesses the frequency and quality of social contact or engagement with friends and family members. LSNS uses a 0 (*none*) to 5 (*nine or more*) Likert-type scale. An example from the questionnaire includes “*How many of your friends do you see or hear from at least once a month?*” (Lubben, 1998). Composite scores are calculated by summing the scores for all six questions, scores range from 0 to 30. Higher scores indicate more social engagement and lower social isolation. LSNS has demonstrated adequate levels of reliability (Lubben, 1998).

#### Social interaction anxiety scale

In the present study, straightforward version of the Social interaction anxiety scale (SIAS) was used. In the straightforward version as opposed to the original 20-item SIAS, only the straightforward (negatively worded) items are used. Rodebaugh et al., (2007) advise using only the straightforward 17-items as they are the better indicators of social interaction anxiety, whereas the three reverse-coded (positively worded) questions fall more closely into the category of extraversion. SIAS uses a 0 (*Not at all characteristic of me*) to 4 (*Extremely characteristic of me*) Likert-type scale (Brown et al., 1997). The scale assesses an individual’s thoughts, emotions, and behaviors in social settings. An example item includes “*When mixing socially, I am uncomfortable*.” Composite scores are calculated by adding the scores for all 17 questions, total scores range from 0 to 68. Higher scores suggest higher social interaction anxiety. Previous research has shown excellent reliability and construct validity for the straightforward SIAS (Brown et al., 1997).

#### Centre for epidemiological studies – depression

The Centre for epidemiological studies – depression (CES-D) is a 20-question measure of depressive symptomology. It uses a 0 (*rare or none of the time*) to 3 (*Most or all of the time*) Likert-type scale (Radloff, 1977). CES-D assesses how an individual may have felt or behaved over the past week (e.g., *I felt that I could not shake off the blues even with help from my family or friends*). Total scores are calculated by reverse coding the positively worded items and summing the scores for all individual items. Composite scores span from 0 to 60, with higher score indicating higher depressive symptomology over the last week. Previous studies demonstrate excellent internal reliability (Radloff, 1977).

#### Health literacy scale

##### Health literacy questionnaire

Health literacy questionnaire (HLQ) has 44 questions and includes nine independent and conceptually different domains or scales. Together these nine scales offer insight into the individual’s knowledge of, engagement with, and application of health-related information and services (Osborne et al., 2013). The nine distinct domains are as follows: (1) Feeling understood and supported by healthcare providers; (2) Having sufficient information to manage my health; (3) Actively managing my health; (4) Social support for health; (5) Appraisal of health information; (6) Ability to actively engage with healthcare providers; (7) Navigating the healthcare system; (8) Ability to find good health information; and (9) Understanding health information well enough to know what to do.

Each scale consists of four to six questions. Domains one to five use a 1 (*strongly disagree*) to 4 (*strongly agree*) Likert-type scale. The remaining four scales employ a 1 (*cannot do*) to 5 (*very easy*) anchor type. Nine separate scores for each domain are calculated by averaging the values (responses) for questions within each of the scales, possible scores for the first five scales are 1 to 4, for domains six to nine, scores can range from 1 to 5. Higher domain scores are indicative of superior understanding and use of health-related services and information (i.e., higher health literacy) for that particular scale. There are no aggregate or total scores for the nine scales as doing so could under- or over-estimate an individual’s particular needs in specific health literacy domains (Osborne et al., 2013). HLQ has demonstrated strong psychometric properties and has been validated for use across diverse settings and populations [e.g., clinical, home, community care, adults 18 to 64 years and over; (Osborne et al., 2013; Elsworth et al., 2016; Kolarcik et al., 2017; Richtering et al., 2017)].

#### Measures for health-related factors

##### Insomnia severity index

Insomnia severity index (ISI) is self-report scale measuring symptoms of insomnia or poor sleep over a fortnight. ISI includes seven items and employs a 0 (*no problem*) to 4 (*very severe problem*) Likert-type scale. An example includes “*To what extent do you consider your sleep problem to INTERFERE with your daily functioning?*” (Morin et al., 2011). Total scores are calculated by adding the scores individual items, scores range from 0 to 28. Higher scores are indicative of more sleeping difficulties. Previous studies demonstrated very strong internal consistency [Cronbach’s *α* ranging from 0.90 to 0.91; (Morin et al., 2011)].

##### Physical health questionnaire

The Physical health questionnaire (PHQ) is a brief self-report measure of somatic health symptoms. The PHQ is a revised version of the health scale created by Spence et al., (1987). PHQ includes 14 questions and four subscales (Schat et al., 2005). The four subscales represent the following four facets of physical health symptoms: sleep difficulties, headaches, gastrointestinal and respiratory tract issues. PHQ employs a 7-point frequency scale, an example includes “*How often have you suffered from an upset stomach (indigestion)?.*” Individual scores can either be calculated for four physical health symptom scales by summing the scores for the corresponding scale questions. Otherwise, as performed in this study, a total somatic health score can be calculated by summing the scores for all four physical health symptom scales. Scores ranged from 7 to 98 with higher scores indicating more somatic health issues. The scale has good psychometric properties (Schat et al., 2005).

##### Diet quality tool

The Diet quality tool (DQT) includes 13 questions which evaluate quality of respondent’s dietary habits (e.g., vegetable and fruit intake, saturated or total fat intake). DQT employs a 0 to 10 type anchor, where a score of 10 is indicative of higher diet quality (i.e., the participant is satisfactorily following the nutritional guidelines to prevent cardiovascular disease) (O’Reilly and McCann, 2012). Nine questions of the DQT include category/quality of food groups consumed, such as types of bread, spreads, pasta/noodles/rice, fish, fat on meats, breakfast foods, milk, salt use in cooking and meals. The four remaining questions assess the quantity of food consumed (e.g., fruit and vegetable intake, quantity of high-fat sweet and savory foods products consumed). Composite scores are calculated by adding answers for each item, total scores span from 0 to 130, higher scores suggesting a higher quality of diet (O’Reilly and McCann, 2012).

##### The Massachusetts general hospital cognitive and physical functioning questionnaire

Cognitive and physical functioning questionnaire (CPFQ) was developed to be a brief measure of cognitive and executive dysfunction in mood and anxiety disorders (Fava et al., 2009). It assesses cognitive and physical functioning over a one-month period. Each CPFQ item is answered with a 6-point Likert-type scale, ranging from 1 (*greater than normal*) to 6 (*totally absent*) (Fava et al., 2009). An example includes “*How has your ability to focus/sustain attention been over the past month?.*” CPFQ has previously shown good test–retest reliability and other psychometric properties (Fava et al., 2009).

##### Perceived general health

Perceived general health was measured using a single question “In general would you say that your health is excellent, very good, good, fair or poor?” on a 1 to 5 (poor to excellent) scale. Higher scores are indicative of higher perceived general health.

##### EUROHIS – Quality of life scale

Quality of life scale (QOL) is an 8-item scale which assess overall quality of life, general health, energy, daily living activity, self-esteem, social relationships, home life, and finances of an individual over a two-week period. QOL is derived from the WHOQOL-BREF and employs a 1 to 5 Likert-type scale. Previous research has demonstrated good internal consistency (Cronbach’s *α* = 0.83) and satisfactory convergent and discriminant validity for QOL (Schmidt et al., 2005).

##### Perceived stress scale-4

This four-item scale of stress was derived from the original 14-item perceived stress scale (PSS). PSS uses a 5-point Liker-type anchor, ranging from 0 (*never*) to 4 (*very often*). It includes both positively and negatively worded questions which evaluate a individuals experience of stressful events over the last month (e.g., “*In the last month, how often have you felt that you were unable to control the important things in your life?*”). PSS is negatively correlated perceived health, social support, being male, and older age (Warttig et al., 2013). Research has demonstrated good reliability and psychometric properties for PSS (Warttig et al., 2013).

##### Positive and negative affect scale

The Positive and negative affect scale (PANAS) short form measures positive (e.g., excited) and negative (e.g., scared) affect over a one-week period. Affect is assessed through 10 items on a 5-point Likert-type scale, ranging from 1 (*very slightly or not at all*) to 5 (*extremely*). PANAS has demonstrated good internal consistency, convergent, and discriminant validity in previous literature (Watson et al., 1988).

### Statistical technique

The data were analyzed using IBM Statistical Package for Social Sciences (SPSS) Version 27.0. A total of 521 participants were included in this study. The data cleaning, screening, and assumption testing information for the statistical techniques used in present study are included in Supplementary material. In the present study, an alpha value less than or equal to 0.05 is used as a cut-off for statistical significance.

To investigate the first aim - the unique contribution of loneliness on health literacy and health-related factors among young adults, we used Hierarchical Multiple Regression, where the predictor variable was loneliness. Outcome variables included nine health literacy scales and various health-related factors (i.e., weight, BMI, somatic health, perceived general health, sleep, diet, alcohol use, physical activity, cognitive and physical functioning, perceived stress, quality of life, positive affect, negative affect). Further, guided by previous empirical research, several covariates were included – age, gender, chronic conditions, scores for social anxiety [covaries with loneliness; (Cacioppo et al., 2010; Trémeau et al., 2016; Abbott et al., 2018)], scores for depressive symptomology [loneliness is often a precursor for depression; (Cacioppo et al., 2010; Stickley and Koyanagi, 2016)], and scores for social isolation [construct oftentimes interchangeably and synonymously used with the term loneliness; (Abbott et al., 2018)]. For all models, in the first step (Model 1) – age, gender, chronic conditions were entered. In step two (Model 2) social anxiety scores were included. In step three (Model 3), depressive symptomology scores were entered. Step four included social isolation scores (Model 4). In the final step (Model 5) loneliness scores were entered to ascertain its unique contribution to various health-related factors and health literacy outcomes among young adults.

To examine our second aim - how social isolation may differ from loneliness in its impact on health literacy and health-related factors of young adults, we utilized Hierarchical Multiple Regression. For these analyzes, the predictor variable was social isolation, outcome variables included nine health literacy scales and various health-related factors. Covariates included age, gender, chronic conditions, depressive symptomology, social anxiety, and loneliness. For all models, in the first step (Model 1) - age, gender, chronic conditions were entered. In step two (Model 2) social anxiety scores were included. In step three (Model 3), depressive symptomology scores were entered. Step four included loneliness scores (Model 4). In the final step (Model 5) social isolation scores were entered to ascertain how it may differ from loneliness in regards to its impact on health literacy and different health-related factors of young adults.

## Results

A complete breakdown of sample characteristics is presented in Table 2. Percentages are included for categorical variables, mean and standard deviation (SD) are included for continuous variables. The Chi squared goodness of fit test for gender was non-significant, suggesting that the sample was not biased (test details and results are included in the Supplementary material).

**Table 2:** Sample characteristics.

	Percentage/mean (SD)
Age (years)	25.2 (5.0)
Gender	
Female	76.6
Male	22.6
Other (non-binary)	0.8
English as a first language	
Yes	88.8
No	11.2
Marital status	
Single/Never married	49.0
Domestic/Defacto relationship	35.1
Married	12.9
Separated/divorced/other	3.0
Ethnicity	
White (includes Caucasian, European Australian)	71.7
Asian Australian or Asian (includes Indian, Indian Australian)	13.5
Aboriginal or Torres Strait Islander	0.8
African Australian	1.2
Hispanic or Latino	2.0
Pacific Islander	0.6
Multiple board categories/not specified	10.3
Sexual orientation	
Heterosexual	80.2
Homosexual (gay/lesbian)	3.6
Bisexual	10.6
Pansexual/Asexual/not specified	5.6
Level of education	
Undergraduate or Bachelor’s degree, diploma, graduate certificate	39.5
Postgraduate or Master’s degree	10.2
TAFE or trade school	12.8
Secondary school	36.9
Primary school	0.6
Living status	
Living with family/housemates/university/relatives	92.8
Living alone	7.2
Smoking status	
Life long non smoker	75.6
Ex-smoker/current smoker	24.4
Chronic conditions	
No chronic condition	16.5
At least have one chronic condition	83.5

*N* = 521.

The descriptive statistics and internal reliability values (Cronbach’s *α*) for all scales used in current study are presented in Table 1.

Correlation matrix for predictors variables and covariates (loneliness, social isolation, depressive symptomology, social anxiety) are displayed in Table 3. There were significant and large associations between loneliness, and social isolation, social anxiety, depressive symptomology, these directions were as expected.

**Table 3:** Correlational matrix for predictor variables and covariates.

		1	2	3	4
1	Loneliness	–	−0.56**	0.56**	0.66**
2	Social isolation	–	–	−0.37**	−0.36**
3	Social anxiety	–	–	–	0.55**
4	Depressive symptomology	–	–	–	–

*N* = 521. Significant *p*-values are bolded, **p < 0.001.

For the first aim, hierarchical regression results, including the *R*^2^ change values (i.e., unique variance accounted by loneliness in the dependent variable) for all outcome variables are presented in Table 4. After controlling for age, gender, chronic conditions, social anxiety, depressive symptomology, and social isolation, increased loneliness was associated with increase in several health literacy domains and health-related factors. Higher loneliness was associated with poorer health literacy among young adults. For examples, those with increased feelings of loneliness also reported lower scores for feeling understood and supported by healthcare providers, poorer social support for health, lower appraisal of health information, poorer ability to find good health information, and lower understanding of health information among young adults. Further, higher loneliness was associated with higher perceived stress and increased negative affect. Increase in loneliness also predicted poorer quality of life among young adults.

**Table 4:** Hierarchical multiple regression models for unique association between loneliness and health-related factors and health literacy in young adults.

Outcome variables	Model 1		Model 2		Model 3		Model 4		Model 5		
	β	*p*	β	*p*	β	*p*	β	*p*	β	*p*	*R*^2^ change
Health related factors											
Weight	0.10	0.014	0.1	0.01	0.03	0.55	0.05	0.35	−0.04	0.56	0.001
Body mass index	0.1	0.02	0.05	0.22	0.07	0.2	−0.009	0.86	0.007	0.91	0.000
Somatic health	0.23	<0.001	0.4	<0.001	0.44	<0.001	0.08	0.05	0.03	0.57	0.000
Perceived general health	−0.19	<0.001	−0.3	<0.001	−0.38	<0.001	0.06	0.18	−0.08	0.18	0.003
Sleep	0.17	<0.001	0.36	<0.001	0.49	<0.001	0.03	0.54	−0.009	0.88	0.000
Diet	0.02	0.6	−0.11	0.02	−0.13	0.02	−0.03	0.54	0.05	0.47	0.001
Alcohol use	0.02	0.66	−0.03	0.55	0.05	0.39	0.15	0.001	−0.03	0.69	0.000
Physical activity	−0.08	0.07	−0.08	0.07	−0.13	0.004	−0.05	0.4	−0.07	0.34	0.002
Cognitive and physical functioning	0.17	<0.001	0.38	<0.001	0.61	<0.001	0.07	0.04	0.006	0.9	0.00
Perceived stress	0.2	<0.001	0.43	<0.001	0.59	<0.001	−0.01	0.77	0.21	<0.001	0.018
Quality of life	−0.18	<0.001	−0.43	<0.001	−0.69	<0.001	0.15	<0.001	−0.21	<0.001	0.017
Positive affect	−0.13	0.003	−0.28	<0.001	−0.5	<0.001	0.06	0.17	−0.07	0.22	0.002
Negative affect	0.13	0.003	0.46	<0.001	0.68	<0.001	0.08	0.02	−0.14	0.002	0.008
Health literacy scales											
Feeling understood and supported by healthcare providers	0.07	0.11	−0.23	<0.001	−0.25	<0.001	0.11	0.02	−0.19	0.004	0.014
Having sufficient information to manage my health	−0.006	0.9	−0.26	<0.001	−0.24	<0.001	0.09	0.05	−0.12	0.06	0.006
Actively managing my health	0.002	0.97	−0.29	<0.001	−0.26	<0.001	0.08	0.09	−0.11	0.09	0.005
Social support for health	−0.06	0.18	−0.33	<0.001	−0.38	<0.001	0.31	<0.001	−0.4	<0.001	0.066
Appraisal of health information	0.09	0.06	−0.28	<0.001	−0.16	0.002	0.01	0.79	−0.17	0.009	0.012
Ability to actively engage with healthcare providers	<0.001	0.99	−0.37	<0.001	−0.25	<0.001	0.13	0.004	−0.11	0.08	0.005
Navigating the healthcare system	−0.07	0.12	−0.32	<0.001	−0.27	<0.001	0.06	0.19	−0.06	0.31	0.002
Ability to find good health information	0.05	0.28	−0.31	<0.001	−0.23	<0.001	0.02	0.61	−0.13	0.046	0.070
Understand health information well enough to know what to do	0.08	0.09	−0.31	<0.001	−0.25	<0.001	0.02	0.64	−0.13	0.04	0.007

*N* = 521. Hierarchical Multiple Regression analyzes results. β = Standardized regression parameter estimates. β values specified in the table are for the last predictor/control variable entered in each model (italicized here). Predictors entered: Model 1 – Age, gender, chronic conditions; Model 2 – Age, gender, chronic conditions, social anxiety; Model 3 – Age, gender, chronic conditions, social anxiety, depressive symptomology; Model 4 – Age, gender, chronic conditions, social anxiety, depressive symptomology, social isolation; Model 5 – Age, gender, chronic conditions, social anxiety, depressive symptomology, social isolation, loneliness. Significant *p*-values are bolded only for unique contribution of loneliness on health-related factors and health literacy (Model 5), *p* < 0.05.

For the second aim, hierarchical regression results, including the *R*^2^change values (i.e., unique variance accounted by social isolation in the dependent variable) for all outcome variables are presented in Table 5. After controlling for age, gender, chronic conditions, social anxiety, depressive symptomology, and loneliness, increase in social isolation was associated with increase in somatic health complaints and alcohol use among young adults. Increased social isolation was associated with lower cognitive and physical functioning. However, note this association was only marginally statistically significant (*p* = 0.058). Those with higher experience of social isolation also reported lower quality of life and lower health literacy in one domain (i.e., social support for health).

**Table 5:** Hierarchical multiple regression models for how social isolation may differ from loneliness in its impact on health-related factors and health literacy among young adults.

Outcome variables	Model 1		Model 2		Model 3		Model 4		Model 5		
	β	*p*	β	*p*	β	*p*	β	*p*	β	*p*	*R*^2^ change
Health related factors											
Weight	0.1	0.02	0.03	0.55	0.05	0.35	−0.05	0.38	0.03	0.62	0.000
Body mass index	0.1	0.02	0.05	0.22	0.07	0.2	0.01	0.86	−0.006	0.91	0.000
Somatic health	0.23	<0.001	0.4	<0.001	0.44	<0.001	−0.02	0.74	0.09	**0.045**	**0.005**
Perceived general health	−0.19	<0.001	−0.3	<0.001	−0.38	<0.001	−0.1	0.07	0.03	0.53	0.001
Sleep	0.17	<0.001	0.36	<0.001	0.49	<0.001	−0.02	0.69	0.02	0.63	0.000
Diet	0.02	0.6	−0.11	0.02	−0.13	0.02	0.06	0.36	−0.01	0.81	0.000
Alcohol use	0.02	0.67	−0.03	0.6	0.05	0.39	−0.11	0.08	0.14	**0.006**	**0.014**
Physical activity	−0.08	0.07	−0.13	0.004	−0.05	0.4	−0.11	0.08	0.08	0.14	0.004
Cognitive and physical functioning	0.17	<0.001	0.38	<0.001	0.61	<0.001	0.04	0.44	0.08	0.058	0.004
Perceived stress	0.2	<0.001	0.43	<0.001	0.59	<0.001	0.177	<0.001	0.06	0.13	0.002
Quality of life	−0.18	<0.001	−0.43	<0.001	−0.69	<0.001	−0.25	<0.001	0.08	**0.03**	**0.004**
Positive affect	−0.13	0.003	−0.28	<0.001	−0.5	<0.001	−0.09	0.09	0.03	0.48	0.001
Negative affect	0.13	0.003	0.46	<0.001	0.68	<0.001	−0.16	<0.001	0.03	0.42	0.001
Health literacy scales											
Feeling understood and supported by healthcare providers	0.07	0.11	−0.23	<0.001	−0.25	<0.001	−0.21	<0.001	0.05	0.36	0.001
Having sufficient information to manage my health	−0.006	0.9	−0.26	<0.001	−0.24	<0.001	−0.15	0.01	0.05	0.33	0.002
Actively managing my health	0.002	0.97	−0.29	<0.001	−0.26	<0.001	−0.13	0.024	0.04	0.42	0.001
Social support for health	−0.06	0.18	−0.33	<0.001	−0.38	<0.001	−0.5	<0.001	0.18	**<0.001**	**0.021**
Appraisal of health information	0.09	0.06	−0.28	<0.001	−0.16	0.002	−0.14	0.014	−0.05	0.38	0.001
Ability to actively engage with healthcare providers	0.000	0.99	−0.37	<0.001	−0.25	<0.001	−0.16	0.005	0.09	0.06	0.006
Navigating the healthcare system	−0.07	0.12	−0.32	<0.001	−0.27	<0.001	−0.09	0.14	0.04	0.46	0.001
Ability to find good health information	0.05	0.28	−0.31	<0.001	−0.23	<0.001	−0.12	0.04	−0.02	0.69	0.000
Understand health information well enough to know what to do	0.08	0.09	−0.31	<0.001	−0.25	<0.001	−0.12	0.04	−0.2	0.64	0.000

*N* = 521. Hierarchical Multiple Regression analyzes results. β = Standardized regression parameter estimates. β values specified in the table are for the last predictor/control variable entered in each model (italicized here). Predictors entered: Model 1 – Age, gender, chronic conditions; Model 2 – Age, gender, chronic conditions, social anxiety; Model 3 – Age, gender, chronic conditions, social anxiety, depressive symptomology; Model 4 – Age, gender, chronic conditions, social anxiety, depressive symptomology, loneliness; Model 5 – Age, gender, chronic conditions, social anxiety, depressive symptomology, loneliness, social isolation. Significant *p*-values are bolded only for unique contribution of social isolation on health-related factors and health literacy (Model 5), *p* < 0.05.

## Discussion

Results from the present study extent previous empirical literature on unique influence of loneliness on health literacy and health-related factors in young adults, after controlling for co-occurring social and mental health symptoms. The present study also examined how the constructs of social isolation and loneliness may differ in their impact on different health-related factors and health literacy of young adults.

Similar to previous literature regarding the negative influence of loneliness on health literacy among older adults (Bennett et al., 2012; Geboers et al., 2016), we found higher levels of loneliness to be associated with lower scores on several health literacy domains in young adults, after controlling for social isolation, depressive symptomology, and social anxiety. For example, young adults with higher loneliness reported lower scores for feeling understood and supported by healthcare providers, poorer social support for health, lower appraisal of health information, poorer ability to find good health information, and lower understanding of health information. These results indicate higher loneliness to be associated with overall lower health literacy among young adults. These findings are particularly concerning as results from the Australian Bureau of Statistics health literacy survey using the HLQ shows over 70 percent of *all* young adults reported higher scores across all nine health literacy scales (Australian Bureau of Statistics, 2018). In other words, young adults, in general, indicated they were adept at understanding, evaluating, and utilizing health information to promote and maintain good health (Australian Bureau of Statistics, 2018). Collectively, these findings suggest that unlike individuals with lower loneliness, young adults with higher loneliness may be at a disadvantage when it comes to understating, appraising, and using health information and health services. This negative relationship between loneliness and health literacy is especially troubling, given the association between lower health literacy and overall poorer health outcomes [i.e., higher hospital re-admission rates; (Mitchell et al., 2012), misunderstanding of medication instructions; (Marvanova et al., 2011), lower adherence to medical treatment; (Miller, 2016), and lower ability to self-managed care; (Geboers et al., 2016)].

Consistent with prior research (Kurina et al., 2011; Ijzerman et al., 2012; Ong et al., 2016; Khalaila and Vitman-Schorr, 2018; Counts and John-Henderson, 2020), and after controlling for concomitant social and mental health factors, we found higher loneliness was associated with higher perceived stress and negative affect, and lower quality of life, this was evident in the adjusted hierarchical regression analysis results included in Table 4. However, contrary to some of the previous research (Hawkley et al., 2009, 2010; Kurina et al., 2011; Fernández-Alonso et al., 2012; Luo and Waite, 2014; Ong et al., 2016; Stickley et al., 2016; Richard et al., 2017; Khalaila and Vitman-Schorr, 2018; Hajek and König, 2019; Counts and John-Henderson, 2020; Griffin et al., 2020; Marziali et al., 2020; Shankar, 2020; Christiansen et al., 2021), we did not find significant relationships between loneliness and several health-related factors including, weight, BMI, somatic health complaints, perceived general health, sleep, diet, alcohol use, physical activity, cognitive and physical functioning, and positive affect, after controlling for the influence of social isolation, depressive symptomology, and social anxiety among young adults. A plausible explanation for the lack of significant relationships between these health-related factors and loneliness could be that some of the predictive and explanatory power attributed to loneliness, in relation to poor health in previous research, may in fact be the influence of social isolation, depressive symptomology, or social anxiety. This premise is supported by the hierarchal regression results presented Table 4 as well as additional analyzes included in Supplementary material, which showcase significant relationships between social anxiety, depressive symptomology, and social isolation, and various health-related factors (e.g., diet quality, somatic health complaints, sleep). Further, as shown in the correlation matrix presented in Table 3, we found strong associations between loneliness, and social anxiety, depressive symptomology, and social isolation, further highlighting the need to adjust for the influence of these social and mental health indicators, when examining the impact of loneliness on health-related factors among young adults.

The findings from present study also shed more light on how loneliness and social isolation may differ in their impact on health-related factors and health literacy of young adults. After controlling for age, gender, chronic conditions, social anxiety, depressive symptomology, and loneliness, higher social isolation was associated with increase in somatic health complaints, namely more respiratory (e.g., cold, flu, bronchitis symptoms) and gastrointestinal tract (e.g., indigestion, nausea, diarrhea) issues, as well as increased complaints of headaches and sleep difficulties. Likewise, higher experience of social isolation was associated with higher alcohol use, lower quality of life, and lower cognitive and physical functioning (marginally significant) among young adults. However, unlike loneliness, increased social isolation was only associated with lower score in one health literacy domain (i.e., social support for health). Together, these findings indicate higher loneliness may more closely affect psychosocial health-related factors (e.g., lower perceived stress, higher negative affect) and health literacy issues, as per associations presented in Table 4. Social isolation on the other hand may influence physical health-related factors of an individual (e.g., somatic health complaints as per associations presented in Table 4), similar to the findings from Beller and Wagner, (2018). Together, these associations suggest that while loneliness and social isolation may be interrelated and share a few superficial characteristics, the mechanisms (psychosocial health and health literacy issues as opposed to physical health issues respectively) in which they influence health literacy and poorer health-related factors of young adults may be different.

### Limitations and future directions for research

Nonetheless, present study was not without limitations. The generalizability of our findings is limited. First, the data collection for this study occurred during the novel coronavirus pandemic when several physical and social distancing restrictions were in place in Australia, especially in the state of Victoria (Department of Health and Human Services Victoria, 2020), where majority of our sample reside. The psychological and social changes associated with the pandemic restrictions may have impacted our findings. Indeed, several studies demonstrate pandemic and its associated social and physical distancing restrictions may have negatively affected the health of many Australians (i.e., psychosocial, physical, and mental health; (Australian Medical Association, 2020; Holmes et al., 2020). Second, a substantial proportion of our sample consisted of first year undergraduate university students, predominantly those who spoke English as a primary language, resided in the state of Victoria, were living with other people, and were Caucasian, and identified as heterosexual females. University student populations are often utilized in psychosocial research as they are an efficient, user-friendly convenience population with lower response bias, administrative cost, and easily recruited (Lucas, 2003; Arnett, 2008). However, it is vital to acknowledge that use of such a sample may not be representative of the general young adult population of Australia, which could result in a more homogenous sample, consequently impacting the generalizability, and by extension, the external validity (McTavish and Loether, 2002) of our findings.

Another limitation of this study was the cross-sectional design used. Use of cross-sectional study design limits our ability to draw causal inferences or ascertain directionality between loneliness and its association with health literacy and various health-related factors among young adults. Future research with longitudinal data and a more heterogeneous young adult population could provide a deeper insight into the strength, causality, and directionality of the unique relationship between loneliness and health literacy and different health-related factors among young adults. Despite these limitations, findings from the current study highlight an information gap and investigate the impact of loneliness on health literacy and various health-related factors among young adults, after accounting for the influence of other concomitant variables. We also demonstrate how loneliness and related constructs such as social isolation may differ in their impact on heath literacy and health-related factors of young adults.

## Conclusion

Loneliness is a relatively common experience which has universally been identified as a growing public health concern with a detrimental impact on health. While traditional research focused on the impact of loneliness on older adults, recent studies demonstrate younger adults are equally as susceptible to the ill effects of loneliness. Our findings show increased loneliness is associated with decrease in several health literacy domains (e.g., poorer social support for health, lower appraisal of health information) and health-related factors among young adults, even after controlling for co-occurring social and mental health symptoms. Additionally, we highlight the importance of controlling for concomitant social and mental health symptoms such as social anxiety, depressive symptomology, and social isolation when investigating the impact of loneliness on health-related factors. Further, we found loneliness and social isolation may in fact differ in the way they impact health literacy and various health-related factors in young adults. However, some considerations need to be made when interpreting our findings, use of cross-sectional study design and primarily female student population limits our ability to make causal inference and generalize our findings across different populations. Nevertheless, findings from the current study contribute to the growing literature on the impact of loneliness on health literacy and various health-related factors among young adults. A better understanding of the impact of loneliness on these health-related issues can inform the development of appropriate public health strategies to facilitate access to health services and health-related information among young adults.

## Data availability statement

The raw data supporting the conclusions of this article will be made available by the authors, without undue reservation.

## Ethics statement

The studies involving human participants were reviewed and approved by Ethical approval was granted by Swinburne University Human Research Ethics Committee (approval number 20202950-4247). The patients/participants provided their written informed consent to participate in this study.

## Author contributions

SV collected the data, performed data analysis, and wrote the manuscript as part of their PhD dissertation. NE, ML, and EL conceptualized and developed the study as well as provided supervision to SV throughout recruitment, data analysis, and manuscript writing. All authors contributed to the article and approved the submitted version.

## Conflict of interest

The authors declare that the research was conducted in the absence of any commercial or financial relationships that could be construed as a potential conflict of interest.

## Publisher’s note

All claims expressed in this article are solely those of the authors and do not necessarily represent those of their affiliated organizations, or those of the publisher, the editors and the reviewers. Any product that may be evaluated in this article, or claim that may be made by its manufacturer, is not guaranteed or endorsed by the publisher.
