# Supplementary material for: Evaluating the impact of loneliness and social isolation on health literacy and health-related factors in young adults
Source: Front Psychol. 2023 Jan 27;14:996611. doi: 10.3389/fpsyg.2023.996611 (PMC9911678; doi:10.3389/fpsyg.2023.996611)
Supplement: Supplementary file 1 [file Data_Sheet_1.docx]

**Supplementary Material**

**Additional materials**

Weight and Body Mass Index (BMI) - Participants self-reported their height and weight measurements including the metric used (for weight - kilograms or pounds and for height - centimeters or feet and inches). BMI values were calculated from the weight and height measurements using the formula BMI = Kilograms/meter ^2^.

Physical Activity - We asked participants to self-report their physical activity levels by asking them questions about the intensity (light, medium or high/vigorous) and the frequency of daily physical activity undertaken (e.g., *“How many days per week do you do* *light/medium/vigorous physical activities for at least 30 minutes?”).* We also provided definitions and examples of light, medium or high/vigorous intensity physical activity in the survey question for their edification. Scores for physical activity were calculated by creating dummy variables and assigning ‘values’ for intensity of physical activity (light = 1, medium = 2, vigorous = 3), and then multiplying the corresponding intensity ‘value’ with frequency of use per week (range 0 to 7). Scores ranged from 0 to 21, with higher scores indicating higher physical activity levels.

Alcohol Use - We asked participants to self-report their alcohol use by asking them whether they drank alcohol (intensity), possible responses included *-* never, rarely (less than once per week), regularly (at least once a week). Further, we asked them to report the number of standard drinks consumed per week (pictures depicting standard drink volumes for several alcohol beverages were included in the survey question). Scores for alcohol use were calculated by creating dummy variables and assigning ‘values’ for intensity of alcohol use (never = 0, rarely (less than once per week) = 1, regularly (at least once a week) = 2), and then multiplying the corresponding intensity ‘value’ with frequency of use (ranging from 1 standard drink per week to more than 10 (coded as 11)) per week. Scores ranged from 0 to 22, with higher scores indicating higher alcohol use.

Chronic conditions - We asked participants to indicate whether they had been previously diagnosed with any of the following conditions, including high blood pressure, high cholesterol, heart palpitations/murmurs/arrhythmias, Thyroid disease, Diabetes, Celiac disease, heart burn/reflux disease, lung disease, liver disease, kidney disease, Asthma, heart disease, sleep apnoea, menstrual irregularity, Polycystic Ovarian Syndrome, Psychiatric disorders (e.g., depression, anxiety, panic disorder, eating disorder or any other), Autoimmune disorders, Cancer, allergies, and none of the above. For the data included in Table 1 - If participants indicated at least one chronic condition, they were included in the ‘Atleast one chronic condition’ category. As a covariate variable, they were included in the regression model as number of chronic conditions per participants. If a participant indicated they were diagnosed with high blood pressure, heart disease, and anxiety disorder -they were given a score of three. Score ranged from 0 to 19, with higher scores here indicating presence of more chronic conditions.

**Data analytic technique**

A total of 802 adults (i.e., individuals residing in Australia at the time of the survey) completed the original online survey conducted as part of the larger study. Of which 407 were community members and 395 were first year undergraduate Swinburne University students. Two hundred and fifty-four participants had at least some incomplete survey responses (i.e., 32% of responses had at least some values missing). In order to obtain a complete data set, we employed multiple imputation (Markov Chain Monte Carlo method with 10 imputation models and 50 iterations using predictive mean matching; [1, 2]) for all continuous variables prior to analyses. Sensitivity analysis indicated missing data and multiple imputation did not affect the observed pattern of associations. Multiple imputation method yielded 700 complete set of responses. We further deleted 16 responses which were either significant univariate (assessed using *z*-scores, skewness and kurtosis absolute values) or a multivariate outlier (assessed using Mahalanobis distance; [3]). As mentioned previously, the data used in the current study was a subset of a larger study. As this study is focusing on young adults, only participants between the ages of 18 years and 35 years were included in the dataset. The final data set used for all analysis performed in this paper consisted of 521 participants. We conducted Chi-square goodness of fit test for ascertain gender bias in our sample, and the results were non-significant χ^2^(1) = 2.65, *p* = .10. Guided by Fowler *et al.* [4] we specified a gender distribution of 74% females and 26% males as majority of our participants were first year undergraduate Psychology students. Results from the Chi-squared goodness of fit test indicate that observed gender ratios for recruited population did not differ from the expected gender ratios.

Hierarchical Multiple Regression were the main analyses used in the current study. All assumption testing for multiple regression analyses were satisfactory. Normality was assessed by visual inspection of histograms and analysing absolute and z-scores values for skewness and kurtosis for all predictors, covariates, and outcome variables. There were some deviations from normality, however, in line with literature [3, 5, 6] given the large sample size, and since we had small to moderate deviations from normality for most of our variables, no transformations were applied. As mentioned above, significant (*p* <0.001) multivariate outliers were deleted. No evidence of multicollinearity and non-independence of errors were found (tolerance value > 0.3 and VIF < 3; Durbin Watson statistic between 1.5 and 2.5 for all outcome/dependent variables; [3]).

**Hierarchical Multiple Regression results for social anxiety and depressive symptomology**

All significant relationships between social anxiety, depressive symptomology, and various health-related factors and health literacy are as follows-

After controlling for age, gender, chronic conditions, social isolation, loneliness, and depressive symptomology, increase in social anxiety predicted increase in somatic health symptoms (more headaches, more gastrointestinal and respiratory track complaints), and sleep issues (insomnia). Conversely, increase in social anxiety predicted decrease in or lower scores in several health literacy domains (Actively managing my health, Appraisal of health information, Ability to actively engage with healthcare providers, Navigating the healthcare system, Ability to find good health information, Understand health information well enough to know what to do) in young adults.

Further, after controlling for age, gender, chronic conditions, social isolation, loneliness, and social anxiety, increase in depressive symptomology predicted increase in somatic health symptoms (more headaches, more gastrointestinal and respiratory track complaints), poorer perceived general health, sleep difficulties (insomnia), lower diet quality, and poorer cognitive and physical functioning. On the other hand, increase in depressive symptomology predicted decrease in different health literacy scales (i.e., Feeling understood and supported by healthcare providers, Having sufficient information to manage my health, Social support for health, Actively managing my health, Ability to actively engage with healthcare providers, Navigating the healthcare system, Ability to find good health information) in young adults. Statistical values for all the regression models with aforementioned results are presented in the excel file embedded below.

**References**

1. Eekhout I, de Boer RM, Twisk JW, de Vet HC, Heymans MW. Missing data: A systematic review of how they are reported and handled. Epidemiology, 2012;23(5):729-32. Available from: doi:10.1097/EDE.0b013e3182576cdb
2. Heymans MW, Eekhout I. Applied Missing Data analysis with SPSS and R studio. 2019. Available from: https://bookdown.org/mwheymans/bookmi/
3. Field A. Discovering statistics using IBM SPSS statistics. London: Sage Publications. 2013.
4. Fowler G, Cope C, Michalski D, Christidis P, Lin L, Conroy J. Women outnumber men in psychology graduate programs. Monitor on Psychology. 2018 Dec;49(11):21.
5. Kim HY. Statistical notes for clinical researchers: assessing normal distribution (2) using skewness and kurtosis. Restor. Dent. Endod*.* 2013;38(1):52-54. Available from: doi:10.5395/rde.2013.38.1.52
6. Tabachnick BG, Fidell LS. Using multivariate statistics. New York: Allyn & Bacon/Pearson Education. 2007.
